# DNA damage repair-related gene signature predicts prognosis and indicates immune cell infiltration landscape in skin cutaneous melanoma

**DOI:** 10.3389/fendo.2022.882431

**Published:** 2022-07-26

**Authors:** Liping Liang, Shijie Mai, Genghui Mai, Ye Chen, Le Liu

**Affiliations:** ^1^ Department of Gastroenterology, State Key Laboratory of Organ Failure Research, Guangdong Provincial Key Laboratory of Gastroenterology, Nanfang Hospital, Southern Medical University, Guangzhou, China; ^2^ Department of Thoracic Surgery, Nanfang Hospital, Southern Medical University, Guangzhou, China; ^3^ Department of Gastroenterology, Integrated Clinical Microecology Center, Shenzhen Hospital, Southern Medical University, Shenzhen, China

**Keywords:** skin cutaneous melanoma, DNA damage repair, immunotherapy, prognostic factor, tumor microenvironment

## Abstract

**Background:**

DNA damage repair plays an important role in the onset and progression of cancers and its resistance to treatment therapy. This study aims to assess the prognostic potential of DNA damage repair markers in skin cutaneous melanoma (SKCM).

**Method:**

In this study, we have analyzed the gene expression profiles being downloaded from TCGA, GTEx, and GEO databases. We sequentially used univariate and LASSO Cox regression analyses to screen DNA repair genes associated with prognosis. Then, we have conducted a multivariate regression analysis to construct the prognostic profile of DNA repair-related genes (DRRGs). The risk coefficient is used to calculate the risk scores and divide the patients into two cohorts. Additionally, we validated our prognosis model on an external cohort as well as evaluated the link between immune response and the DRRGs prognostic profiles. The risk signature is compared to immune cell infiltration, chemotherapy, and immune checkpoint inhibitors (ICIs) treatment.

**Results:**

An analysis using LASSO-Cox stepwise regression established a prognostic signature consisting of twelve DRRGs with strong predictive ability. Disease-specific survival (DSS) is found to be lower among high-risk patients group as compared to low-risk patients. The signature may be employed as an independent prognostic predictor after controlling for clinicopathological factors, as demonstrated by validation on one external GSE65904 cohort. A strong correlation is also found between the risk score and the immune microenvironment, along with the infiltrating immune cells, and ICIs key molecules. The gene enrichment analysis results indicate a wide range of biological activities and pathways to be exhibited by high-risk groups. Furthermore, Cisplatin exhibited a considerable response sensitivity in low-risk groups as opposed to the high-risk incidents, while docetaxel exhibited a considerable response sensitivity in high-risk groups.

**Conclusions:**

Our findings provide a thorough investigation of DRRGs to develop an DSS-related prognostic indicator which may be useful in forecasting SKCM progression and enabling more enhanced clinical benefits from immunotherapy.

## Introduction

Skin cutaneous melanoma (SKCM) is identified as one of the most frequent, belligerent, and life-threatening primary malignant skin cancer usually associated with distant metastasis as well as high mortality (1). In recent years, the most common treatment modalities for SKCM are surgery, chemotherapy, and immunotherapy. Despite, great success in SKCM treatment, a 5-year overall survival (OS) rate among metastatic melanoma patients remains extremely poor which is mainly attributed to late diagnosis, rapid metastasis, and ineffective treatment response (2). Depending on the clinical characteristics of the patient, risk stratification and subsequently individualized treatment therapy based on the degree of risk may help in improved prognosis (3). Nonetheless, the existing tumor staging system is inadequate to effectively forecast the prognosis of SKCM and hence there is an unmet need to find new biomarkers which can predict the prognosis of patients with SKCM.

In recent years, the progress in the field of genomics and bioinformatics has made it possible to discover new biomarkers and drug targets. Researchers have identified many biomarkers for diagnosis, prognosis, and treatment, including noncoding RNAs (lncRNA), microRNAs (miRNAs), and messenger RNAs (mRNAs). Some immune-related markers, for example, are being utilized to assess tumor microenvironment (TME) infiltration patterns and reveal any relationship between TME and clinical properties (4). Furthermore, there are reports where prognosis has been predicted using signatures such as hypoxia, autophagy, m5C or m6A mRNA modification, and lactate metabolism (5, 6).

Researchers have thoroughly examined DNA damage repair (DDR) in the context of tumors and neoplasia where they found defective DDR can induce an accumulation of DNA damage as well as genome instability that lead to tumor occurrence. Exonuclease 5 gene germline mutations have been reported to impair DNA repair resulting in androgen-associated prostate cancer (7). Nonetheless, DNA repair may be associated with vulnerability towards anticancer treatments such as radiation therapy or poly ADP-ribose polymerase (PARP) suppressors during tumor development. In response to ionizing radiation, MAP kinase-ERK kinase 5 (MEK5) has been reported to stimulate the phosphorylation of DNA-PK (8). In breast cancer, a higher threonine tyrosine kinase (TTK) expression is linked with effective homologous recombination-mediated repair and radiation sensitivity (9). All these earlier studies have emphasized on the significance to study various functions of DNA repair genes in cancer.

In previously published reports, SKCM is considered as a belligerent tumor exhibiting significant heterogeneity and high genomic mutations which suggests that subgrouping tumors based on gene expression patterns will be ultimately key to accurately assessing melanoma progression (10, 11). As a result of such subgroupings, more targeted therapy may be devised. There have been many reports on the prognostic and biological importance of genetic changes linked to cancer such as PARP1 (12, 13), NRAS (14), absent in melanoma-1 (AIM1) (15), Methylguanine-DNA Methyltransferase (MGMT) (16) as well as KPNA2 (17) mutations in SKCM. Nevertheless, the functions of DNA repair genes for the maintenance of genomic stability among SKCM are seldom described.

In this research, we have gathered as well as evaluated data retrieved from multiple databases like TCGA (The Cancer Genome Atlas), GTEx (The Genotype-Tissue Expression), and GEO (The Gene Expression Omnibus) to ascertain the DNA repair gene’s potential for prognosis of SKCM patients. Hereby, we have constructed a forecasting model premised on the DRRGs (DNA repair-related genes) expression and assessed distinct tumor immune infiltrating landscapes linked to the gene profiles.

## Materials and methods

### Datasets

For this study, we have retrieved the survival information along with gene expression datasets from the combined TCGA-SKCM dataset (https://portal.gdc.cancer.gov/) which includes around 446 tumor samples for training purposes (**Supplementary Table 1**). SKCM patient’s expression profiles with survival data are retrieved from the GEO database (http://www.ncbi.nlm.nih.gov/geo) with accession number GSE65904 for validation purposes (n = 210, **Supplementary Table 2**).This study also included 557 non-tumor normal samples from the GTEx dataset (https://gtexportal.org/). DDR gene data (Hallmark DNA Repair Data Set) was downloaded from MSigDB database.(http://www.gsea-msigdb.org/gsea/index.jsp). There is no need for permissions from the ethics committee as the data were obtained from TCGA, GTEx, and GEO datasets, and have been reported in this work by carefully adopting the publication criteria established by these individual databases.

### Identification of DDR-related genes and development of a prognostic signature in SKCM

We have retrieved prospective DNA repair genes from the TCGA dataset which are substantially linked to the prognosis of SKCM patients using the univariate Cox regression analysis. Following that, we used LASSO (Least absolute shrinkage and selection operator) regression analysis to identify the best prognostic genes while avoiding model overfitting. As a final step, we developed a risk score algorithm using the gene expression levels weighted by regression coefficients from multivariate Cox regression analyses. Each patient’s risk score is computed by using the below-mentioned algorithm:


$$Risk\;score=\sum_{i=1}^{n}\left(Coefi*xi\right)$$

where *Coef_i_* denotes risk coefficients, *x_i_* stands for the expression value of DRRGs (18).

### Assessment and validation of the prognostic signature

The risk score of each patient is sorted (computed as per the aforementioned algorithm) while setting the median risk score as the critical value and based on this, the training and validation cohorts are divided into high- and low-risk cohorts. To distinguish the survival times of the two patient cohorts, the Kaplan-Meier (KM) curve was used. In addition, receiver operating characteristic (ROC) curves are used to evaluate the predictive effect of the signature, where a prediction with an area under the curve (AUC) of greater than 0.60 is considered to have medium accuracy, whereas a prediction with an AUC of greater than 0.75 is considered to have high accuracy. Multivariate and univariate Cox regression analyses were undertaken to investigate if the risk score is independent of other clinical parameters such as age, gender, stage, tumor mutation burden (TMB), tumor purity, cytolytic activity score (CYT), and riskScore. Subsequently, subcohort analysis of a single gene in the DDR-related prognosis model is carried out premised on the clinical features of the patients. Additionally, using TCGA data, we examined the connection between risk scores and clinicopathological characteristics of patients with SKCM. After incorporating the recorded risk scores into the present staging method, we evaluated its usefulness in stratifying risk levels. Premised on the clinical data and risk scores of patients, the “rms” package was utilized to create a nomogram for clinical assessment. We then plotted a calibration to examine the consistency among the forecasted and the actual clinical results and computed the concordance index (C-index) for the nomogram model. The nomogram model’s reliability and the prognostic value of DRRG were verified utilizing an independent dataset (GSE65904).

### Gene set enrichment analysis and single sample GSEA

According to the DRRGs prognostic signature, the functional phenotype between the high- and low-risk cohorts was evaluated by gene set enrichment analysis (GSEA) software. GO gene sets (go.bp.v7.4.symbols.gmt) obtained from the Molecular Signatures Database were employed as the baseline gene set (19). The critical values used in the study included the Nominal p-value < 0.05, FDR < 0.25, and |NES| (Normalized Enrichment Score) > 1. The variation in the expression of immune-associated activities and immune cell infiltration among the patient cohorts was assessed utilizing a single sample gene-set enrichment analysis (ssGSEA).

### Tumor-infiltrating immune cells (TIIC) fraction assessment

We tried to evaluate the immunological infiltration degree of about 22 immune cell types into mixed cell populations based on specific gene expression characteristics among 22 leukocyte subtypes LM22 by using the ‘‘CIBERSORT’’ method as described previously (20). The “pheatmap” program has been utilized to visualize the distribution of immune cells in the two cohorts and then the Wilcoxon rank test was applied to contrast the differences among quantities of immunological infiltrates for the low-and high-risk cohorts while p-values are determined by “vioplot” R package.

### Estimation of the immunoreactivity

We have used the Wilcoxon test to evaluate the expression of PD-1, CTLA4, PD-L1, and TGFB1 among low-as well as high-risk cohorts as key hub immune response biomarkers. In order to predict response to immune checkpoint blockade (ICB) in the TCGA-SKCM dataset, we utilize the immunophenoscore (IPS) retrieved from The Cancer Immunome Atlas (TCIA) (https://tcia.at/home). Anti-CTLA-4 and anti-PD-1 antibody responses are reliably predicted by IPS. Positive correlations between IPS and ICB responses are generally seen; higher scores are associated with greater immunoreactivity (21).

### Statistical analyses

The Student’s *t*-test is being used to compare the differences between the two cohorts. The log-rank method is applied to compare the survival curves for disease-specific survival (DSS) rates from the KM survival analysis. The statistical analysis is carried out using GraphPad Prism (version 8.0) and R software (version 4.0.4). The p-values (<0.05) are considered statistically significant.

## Results

### Identification of prognosis-related DRRGs and construction of a prognostic signature

**Figure 1:**
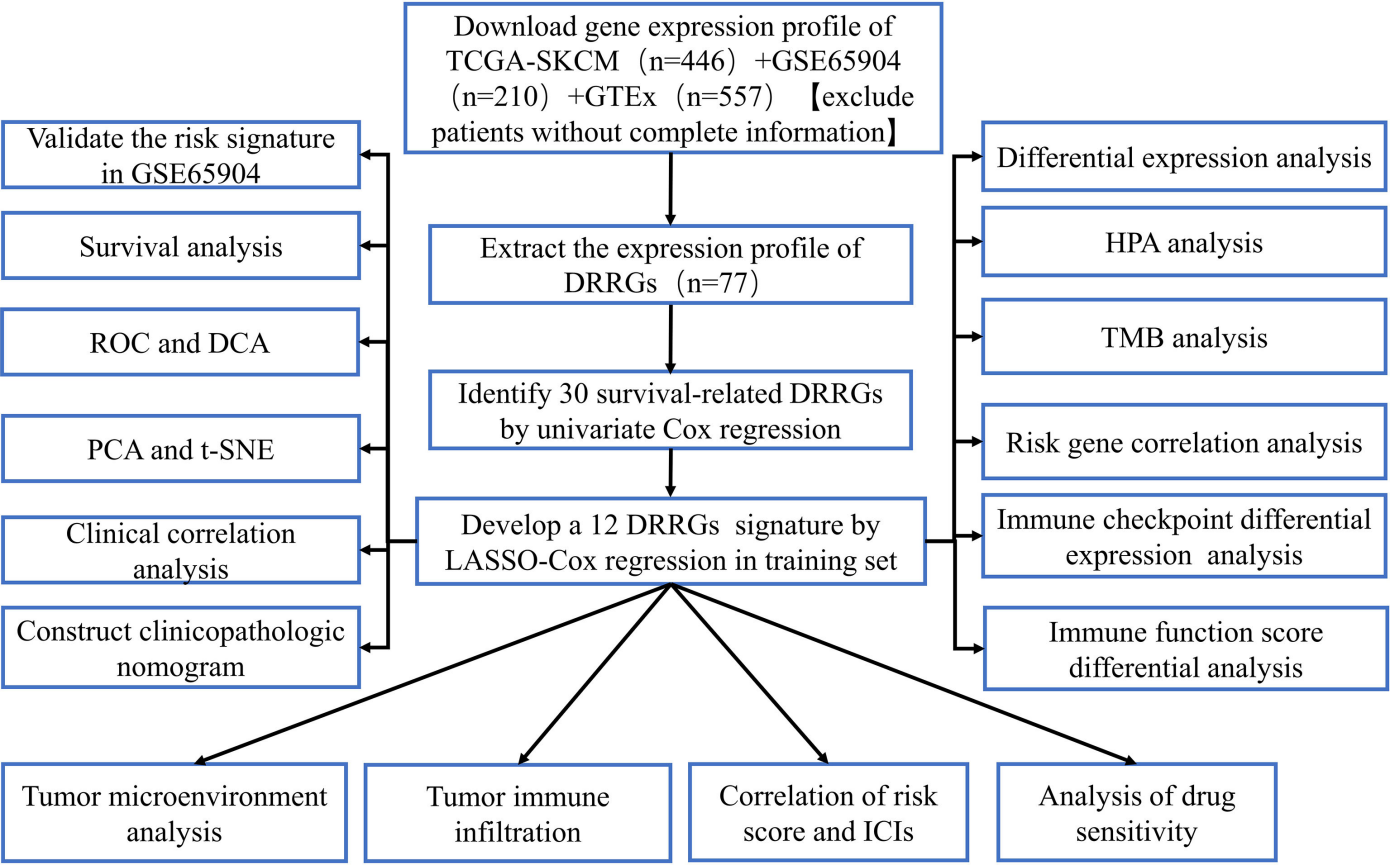
The flow diagram of this study.

**Figure 2:**
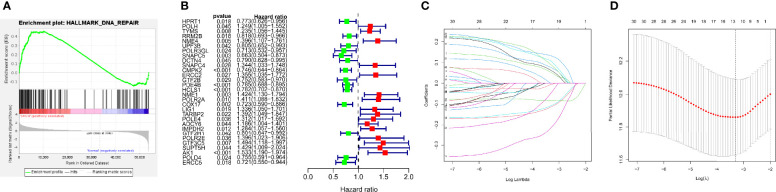
Construction of DRRGs signature. **(A)** Significant enrichment of DNA repair-related pathways in SKCM patients. NES, normalized enrichment score; **(B)** The forest plot of the univariate Cox regression depicted 30 DRRGs associated with SKCM survival; **(C)** Lasso regression for DRRGs in univariate Cox regression; **(D)** Coefficients of selected features denotes the risk coefficient is shown by lambda parameter.

**Figure 1** depicts the workflow design used in this research. We performed GSEA of SKCM and normal tissue samples. The findings demonstrated that SKCM is substantially associated with positive modulation of the DDR response (NES = 1.72, p < 0.01) (**Figure 2A**). And we have found from the TCGA-SKCM training dataset, that the univariate analysis is clearly illustrating the expression of 30 DRRGs being considerably linked to the prognosis of SKCM patients (p < 0.05), and among them, 14 were protective genes (hazard ratio <1), and 16 genes were associated with increased risk (hazard ratio >1) (**Figure 2B**). Subsequently, in order to generate a prognostic signature, the LASSO approach was used to reduce the number of candidate genes based on the minimal penalty parameter (λ) (**Figures 2C, D**). In combination with Multivariate Cox regression analyses, a total of 12 DRRGs (TYMS, SNAPC5, CMPK2, PDE4B, HCLS1, NME1, POLR2A, COX17, LIG1, POLE4, GTF2H1, and AK1) were identified as predictive indicators for patients with SKCM (**Supplementary Table 3**). The DRRGs signature risk model was formulated as: Risk score = (0.053 * TYMS exp.) + (−0.006 * SNAPC5 exp.) + (−0.108 * CMPK2 exp.) + (-0.114 * PDE4B exp.) + (-0.084 * HCLS1 exp.) + (0.076 * NME1 exp.) + (0.107* POLR2A exp.) + (-0.215 * COX17 exp.) + (0.063 * LIG1 exp.) + (0.090 * POLE4 exp.) + (-0.048 * GTF2H1 exp.) + (0.132 * AK1 exp.).

### Prognostic analysis of the 12-gene marker in TCGA-SKCM cohort

**Figure 3:**
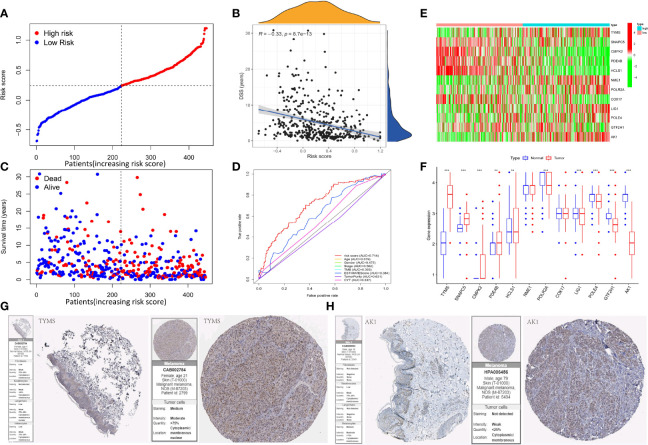
Construction of the DRRGs signature and prognostic analysis. **(A)** Risk score distribution among patients with SKCM; **(B)** Spearman correlation analysis of risk score and disease-specific survival (DSS); **(C)** Survival status of each patient with SKCM. Blue signifies low risk or alive while red signifies high risk or dead; **(D)** Heatmap of gene expression between the high and low-risk cohort; **(E)** Relative gene expression between the high and low-risk cohort; **(F)** AUC values for risk score, age, gender, stage, TMB, ESTIMATE score, tumor purity, and CYT classification; **(G)** Immunohistochemical staining images from The Human Protein Atlas of 2 key genes in SKCM.

We created a prognostic signature by generating a computed risk score premised on the expression of selected 12 important prognostic genes. The specimens are categorized into high- and low-risk cohorts based on the median risk score (**Figure 3A**). The distribution of risk scores, a summary of the survival are shown in figure (**Figures 3B, C**). In addition, a heatmap displaying the expression pattern of each gene was created to illustrate the disparity between the high- and low-risk groups predicted by the prognostic model (**Figure 3E–F**). The risk model exhibits specificity and sensitivity in-consistent with or rather much better than other conventional prognostic variables, as illustrated by the areas under the ROC curve for risk score, age, gender, stage, TMB, ESTIMATEScore, TumorPurity, and CYT classification, which were found to be 0.714, 0.619, 0.475, 0.562, 0.393, 0.384, 0.631, and 0.337 respectively (**Figure 3D**). We have further utilized the human protein atlas immunohistochemistry dataset (www.proteinatlas.org) to evaluate the expression of the DRRGs visually in cancerous and non-cancerous tissue, using this we examined the protein expression of the 2 main genes in SKCM. In tumor tissues, AK1 staining was lower, while TYMS staining was higher (**Figures 3G–H**). Interestingly, all of genes that had protein expression staining in the tumor stromal tissue might influence tumorigenesis and prognosis of SKCM through interstitial components.

### Assessment and validation of the DRRGs prognostic signature

**Figure 4:**
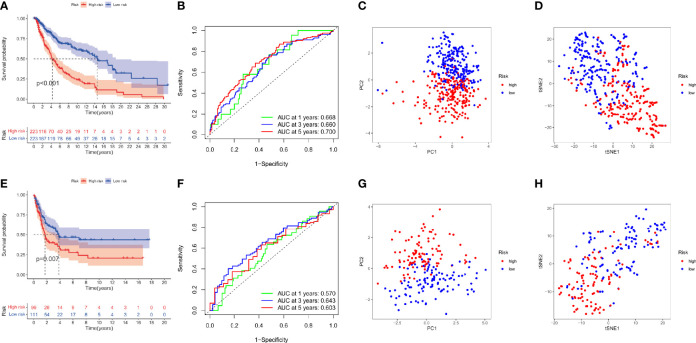
DRRGs signature based on training (TCGA-SKCM) and testing (GSE65904) cohorts. **(A)** KM plot of DSS premised on the risk scores; **(B)** ROC for DSS; **(C)**PCA plot; **(D)** t-SNE analysis in the training cohort (TCGA-SKCM); **(E)** KM plot of DSS premised on the risk scores; **(F)** ROC for DSS; **(G)** PCA plot; **(H)** t-SNE analysis in the test cohort (GSE65904).

The survival analysis is performed to assess the signature profile where the KM curve has shown a dismal prognosis for high-risk patients (**Figure 4A**). The ROC curve effectively revealed the performance of DRRGs prognostic signature in predicting one-, three- and five- survival rates in the TCGA-SKCM dataset as illustrated by AUC values of 0.668, 0.660, and 0.700, correspondingly (**Figure 4B**). The t-SNE analysis and principal component analysis (PCA) revealed that the distribution mode of the two patient cohorts are differerent (**Figures 4C, D**). Moreover, the results from the GSE65904 dataset are verified with a similar risk coefficient and found to agree with the findings from the TCGA-SKCM dataset where the high-risk cohort appeared to have worse results than the low-risk cohort (**Figure 4E**). ROC curves have shown that the AUC for one-, three-, and five-year survival rates in the GSE65904 cohort are 0.570, 0.643, and 0.603, accordingly (**Figure 4F**). Similarly, the t-SNE analysis and PCA plot have illustrated that specimens from 2 risk cohorts are dispersed in 2 routes (**Figures 4G, H**).

### Construction of the nomogram to predict the survival for SKCM patients

**Figure 5:**
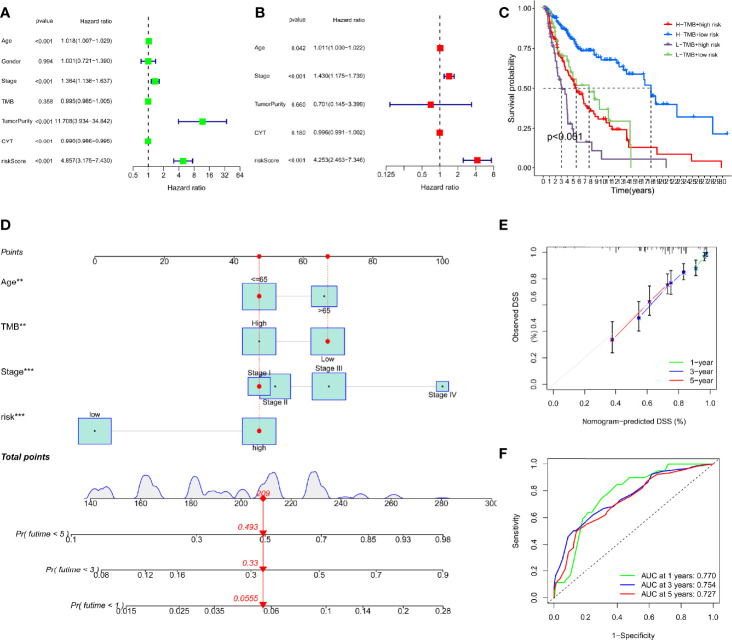
Construction of a nomogram based on the DRRGs signature. **(A)** The univariate Cox analysis illustrated that the age, stage, tumorpurity, CYT and riskScore were statistically distinct; **(B)** The multivariate cox analysis illustrated the age, stage and riskScore were 3 independent predictors of SKCM prognosis; **(C)** Survival curve for patients with distinct TMB and risks; **(D)** The nomogram integrated with the parameters (riskScore, stage, and age) amongst patients from the TCGA cohort; **(E)** Calibration curve of the nomogram at 1, 3, and 5 years; **(F)** AUC values for 1-, 3-, and 5- year survival rates premised on the nomogram.

To ascertain whether the DRRGs signature could be used as an independent predictor variable in SKCM, we have added risk scores and several clinicopathologic characteristics based on the TCGA-SKCM cohort. The results showed the constructed model (riskScore) remained significant through both multivariate and univariate Cox regression analyses (p<0.001, **Figures 5A, B**). When the combination of TMB is used, the prediction performance of the risk score is better than if they are used separately. Furthermore, it is found that the low TMB+high-risk cohort possesses a greater survival risk in the hazard than the other cohort (KM analysis, **Figure 5C**). Nomograms are a way to compress statistical models into a single numerical estimation of probability, such as death or recurrence. They are so widely used for cancer prognosis because they are personalized to the profile of every patient. In our study, a prognostic nomogram integrating clinical characteristics (stage, age, TMB) and the DRRGs-based signature is developed, which can predict the survival likelihood of SKCM patients (**Figure 5D**). The calibration curve further established the hybrid nomogram’s high veracity and reliability (**Figure 5E**). Last but not least, we computed nomogram model scores and ROC analysis based on these scores. The outcomes showed that the model, after incorporating clinical data, appeared to increase AUC values for the TCGA dataset (one- year: 0.770, three-year: 0.754, five-year: 0.727) (**Figure 5F**).

### Characterization of the relationship between risk score and tumor immune ,microenvironment characteristics

**Figure 6:**
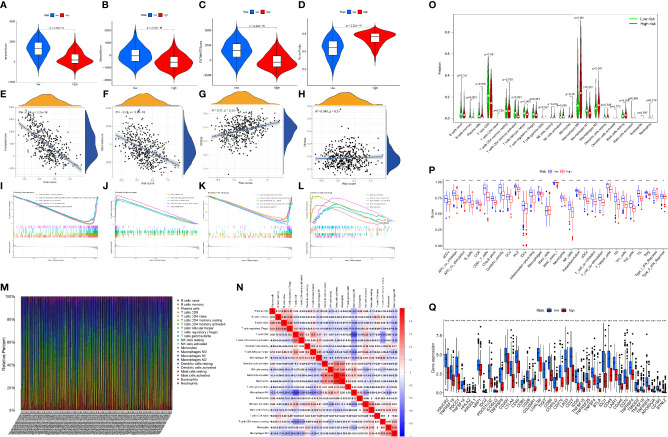
GSEA of SKCM patients premised on the DRRGs prognostic signature. **(A–D)** The expression differences of immune score, stromal score, ESTIMATE score, and tumor purity in high- vs. low-risk cohort; **(E–H)** Spearman correlation analysis of risk score with immune score, stromal score, RNAss, and DNAss; **(I–L)** GSEA outcomes illustrated substantial enrichment of immune-associated biological mechanism in the low-risk patients; **(M)** A Barplot of the 22 immune fractions designated by dissimilar colors in each SKCM sample; **(N)** Correlation heatmap of the ratio of TIICs; **(O)** Wilcoxon test of 22 immune fractions in high- vs. low-risk cohort; **(P)** Link between risk score and immune cell infiltration and related roles through the ssGSEA analysis. The score denotes the immune score, with elevated scores signifying a deeper extent of immune cell infiltration; **(Q)** ICB molecules expressed differently between high- and low-risk groups; (ns, not significant, *P < 0.05, **P < 0.01, and ***P < 0.001).

Researchers have found a link between tumor-infiltrating lymphocytes (TILs) and the growth of cancer, resistance to drugs, and how well treatment works. We also looked at the relationship between the immune-associated score, the risk score (which we got from the R package “ESTIMATE”), the immune cell types and abundance (which we got from the CIBERSORT approach), and the expression levels of ICB-associated genes to see what role the risk score might play in the tumor immune microenvironment (TIME) characterization of SKCM. The findings of this study illustrated that the low-risk cohort exhibited elevated ImmuneScore, StromalScore, and matching ESTIMATEScore as well as reduced TumorPurity than the high-risk cohort (**Figures 6A–D**). Additionally, we noticed a substantial correlation between the risk score and the variables ImmuneScore, StromalScore, and RNAss (**Figures 6E–H**). According to the GSEA, immune-associated biological processes such as CHEMOKINE_SIGNALING_PATHWAY, CYTOKINE_CYTOKINE_RECEPTOR_INTERACTION, HEMATOPOIETIC_CELL LINEAGE, INTESTINAL_IMMUNE_NETWORK_FOR_IGA_PRODUCTION, and LEISHMANIA_INFECTON are significantly enriched in the low-risk cohort (**Figures 6I–L**). These findings suggest that in the low-risk cohort, activating immunomodulatory processes may contribute to an improved prognosis. Also, we created a box plot to exhibit the exact proportions of 22 immune cells based on the CIBERSORT algorithm among all SKCM samples (**Figure 6O**). The correlation analysis of the extent of the 22 immune cells is also shown in **Figure 6N**. The outcomes from the Wilcoxon rank test reveal that the high-risk cohort exhibit reduced infiltration levels of macrophage M1 cells, follicular helper-T cells, CD8+ T-cells, CD4+ memory-activated T-cells, memory B-cells, and plasma cells as compared to the low-risk cohort (**Figure 6O**). This study also demonstrated a substantial relevance between risk scores and the expression of several immunological checkpoint-related genes where expression of all immunological checkpoint-related genes was elevated in the low-risk cohort as compared to the high-risk cohort (**Figure 6P**). Further, the box plots demonstrate variations for immune cell infiltration and related functions among distinct risk sub-cohorts (**Figure 6Q**). We hereby evaluated that the low-risk cohort possesses better cytotoxicity as well as more checkpoint signatures like HLA, CD8+T cells, NK cells, neutrophils, etc. Overall, these outcomes indicate that the DRRGs-based risk characteristics may be to some extent promote a new understanding of the TIME characteristics and immune response of SKCM patients.

### The DDR-related risk signature and mutation profiles

**Figure 7:**
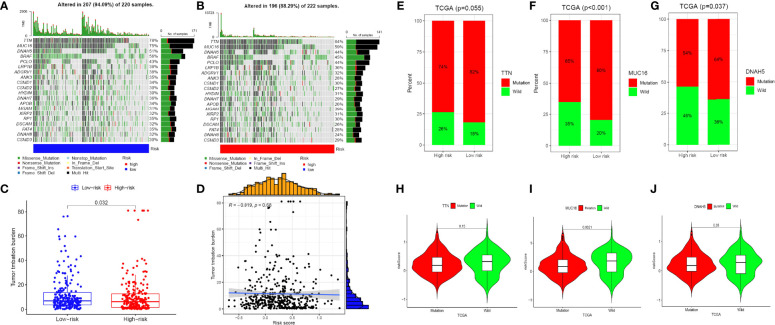
The mutation profile and TMB among low-risk and high-risk groups. **(A, B)** Mutation profile of low-risk and high-risk groups. **(C, D)** The relationship between the immune-related risk signature and TMB. **(E–G)** The proportion of mutation of TTN, MUC16 and DNAH5 in both low-/high-risk groups form the TCGA-SKCM dataset. **(H–J)** Comparison of the risk score between mutation and wild groups.(ns, not significant, *P < 0.05, **P < 0.01, and ***P < 0.001).

The relationship between the mutation profile and the signature was evaluated in TCGA-SKCM patients with available somatic mutation data. The top ten mutated genes in SKCM patients were: TTN, MUC16, DNAH5, BRAF, PCLO, LRP1B, ADGRV1, ANK3, CSMD1, and CSMD2. The most frequently mutated genes in the low-risk and high-risk groups are presented in **Figures 7A, B**. Surprisingly, TMB and the result was statistically significant (p = 0.032; **Figures 7C, D**). In addition, we proposed to investigate the function of gene mutation in risk scores and examined the fraction of mutation genes in both low- and high-risk groups in accordance with the results of somatic mutation data. While TTN mutation was similar in the two different risk groups, we found that MUC16 and DNAH5 mutations were substantially connected with a risk score (**Figures 7E–J**). The fact that there is a correlation between the amount of TMB and the risk score in SKCM, when taken together, suggests that TMB may play an important part in predicting the outcomes of patients.

### Response of high- and low-risk patients to immunotherapy, targeted therapy, and chemotherapy

**Figure 8:**
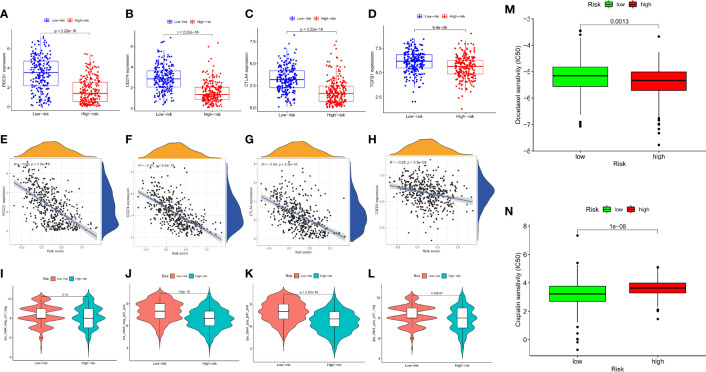
Relationship Between the Prognosis-Associated Immune Signature and Drug Response in SKCM. The differential expression of **(A)** PDCD1, **(B)** CD274, **(C)** CTLA4, **(D)** TGFB1 in the two subgroups, correspondingly; Spearman correlation analysis of risk score and **(E)** PDCD1, **(F)** CD274, **(G)** CTLA4, **(H)** TGFB1; **(I)** IPS score distribution plot; **(J)** IPS-PD1 blocker score distribution plot; **(K)** IPS-CTLA4/PD1 blocker score distribution plot; **(L)** IPS-CTLA4 blocker score distribution plot; **(M)** Boxplot comparing patient response to docetaxel chemotherapy; **(N)** Boxplot comparing patient response to cisplatin chemotherapy. (ns, not significant, *P < 0.05, **P < 0.01, and ***P < 0.001).

We attempted to examine the effects of DRRGs-based signature on TIME of SKCM for which around 4 hub immune checkpoint inhibitors (ICIs)-associated genes (i.e. PDCD1, CD274, CTLA-4, TGFB1) are singled out for additional research. Here an assessment of the connections between risk score and immunological checkpoint gene expression could yield novel therapeutic concepts. The expression patterns of inhibitory receptors (PDCD1, CD274, CTLA4, and TGFB1) are considerably elevated in the low-risk cohort in comparison to the high-risk cohort (**Figures 8A–H**). It is found that there may be a connection between TIIC alterations and the survival time of SKCM patients based on differences between the two cohorts. IPS is a reliable predictor of anti-CTLA-4 and anti-PD-1 antibody responses. Immunophenoscore (IPS) accurately predicts anti-CTLA-4 and anti-PD-1 antibody responses. IPS are typically connected positively with the ICB response. We studied the association between IPS and our DRRGs risk model in TCGA-SKCM, and the results indicate that there was no significant variation in immunophenoscore between risk groups in IPS_CTLA4_neg_PD1_neg (**Figures 8I**). In the low-risk group, IPS-PD1, IPS-CTLA4, and IPS-PD1-CTLA4 blocker scores were higher, indicating greater immunotherapeutic benefits (**Figures 8J–L**). The fact that chemotherapy is also a common treatment method for SKCM prompted us to assess the sensitivity of known anticancer clinical drugs (cisplatin and docetaxel) through the “pRRophetic” R package premised on the assessment of the tumor genes expression level. After calculating the sample’s IC_50_, we found that docetaxel exhibits a considerably higher response sensitivity in high-risk incidents than in low-risk incidents, contrary cisplatin shows higher response sensitivity in low-risk incidents (**Figures 8M, N**).

## Discussion

Due to its high rate of metastasis, invasiveness, and yearly increasing prevalence, SKCM has been reported as the leading cause of skin cancer death worldwide. There are several complicated multistep mechanisms responsible for the onset, progression, and metastasis of SKCM, yet its pathogenesis remains unknown, and there are no effective prognostic indicators for the disease. Therefore, understanding the underlying molecular mechanisms and identifying novel biomarkers is helpful for the prognostic prediction, risk stratification, and therapeutic target of SKCM. The current investigation was designed as a pilot study to find possible biomarkers associated with prognosis and also to test novel research ideas for the future. As a means of guiding individual therapy, prediction models have been investigated for many years. According to reports, models based on tumor gene expression could predict how patients would respond to gemcitabine and fluorouracil (22). Using genetic characteristics, Zhao et al. classified triple-negative breast tumors into different subgroups and evaluated the clinical effect of subtyping-based targeted treatment (23). It is uncommon for SKCM patients to receive individualized treatment based on molecular subtyping.

Research has shown that DNA damage response pathways are critical for correcting and repairing DNA damage, which can prevent cell aging, apoptosis, and carcinogenesis in the long run, as well as ensure activities of daily living (24). Specific DDR pathways include Specifically, DDR is made up of 8 pathways which include: variable DNA synthesis, Fanconi’s anemia, checkpoint factor, non-homologous end ligation, mismatch repair, homologous recombination repair, nucleotide excision repair, and base excision repair (25). Together, these mechanisms could repair DNA damage properly and promptly, maintain genomic integrity, and avoid gene distortion. Recent research has revealed that increased DNA damage and decreased cancer cell DNA repair capacity result in cancer cell genome distortion and that differentiating these cells from normal cells could enhance cancer therapy efficacy (26). DDR genes are cancer-driving and play an important role in clinical and translational medicine, and as a result, they can provide additional treatment options for cancer patients (27). SKCM has been connected to the DDR-related pathway, and the expression of particular DNA repair components has been found to be associated with the prognosis of the patient (28).

Numerous prognostic models premised on immunology, glycolysis, and autophagy genes have been developed, and their predictive usefulness in various kinds of cancer has been examined. Nevertheless, the prognostic value of DNA damage genes in cancer is still debatable. The present study examined the effects of DNA repair genes on SKCM development and patient outcome. Thirty DRRGs were identified using univariate Cox regression analysis, and the best 12 were identified using LASSO-Cox regression analysis (TYMS, SNAPC5, CMPK2, PDE4B, HCLS1, NME1, POLR2A, COX17, LIG1, POLE4, GTF2H1, and AK1). Following the completion of the calculation of risk scores using the risk coefficients, the patients were divided into two distinct groups. According to the examination of the survival data, high-risk patients as per the score appeared to have a dismal prognosis. Additionally, multivariate and univariate Cox regression studies illustrated that the tumor stage and signature were 2 independent prognostic variables. Moreover, the DRRGs prognostic signature was validated by utilizing the independent data set GSE65904.

5-FU has been reported to be an antimetabolite drug that causes cytotoxicity primarily by inhibiting thymidylate synthase (TYMS) resulting in dTMP depletion, compromising DNA synthesis. Patients diagnosed with CRC who were given chemotherapy based on 5-FU to address their condition, have been shown to have higher TYMS expression and lower survival when the insertion (ins) allele or the triple tandem repeat (3R variant) is present (29). Despite the lack of documentation of SNAPC5’s involvement in cancers, the results of this research suggest that further investigation will be needed. There is also evidence that CMPK2 and PDE4B, which are immune checkpoint proteins in cancers, inhibit cell proliferation and induce apoptosis (30, 31). HAX1 is implicated in apoptosis, cell migration, and calcium homeostasis. HAX1 protein partners were identified and their significance in oxidative stress and aggregation was studied (32). NME1/NM23-H1 nucleoside diphosphate kinase is a well-recognized metastasis inhibitor, with NME1 downregulation influencing in situ-to-invasive shift in the process of breast cancer development (33). POLR2A *de novo* germline variant has recently been linked to neurodevelopmental disease. POLR2A-associated developmental disorders are most likely a spectrum of linked, multi-systemic developmental diseases caused by different processes that converge at a single locus (34). The human cytochrome C oxidase assembly protein 17 (Cox17) has been recognized as an essential copper chaperone that facilitates the transfer of copper ions to the mitochondrion. *In vitro* investigations led by Zhao et al. have recently revealed that the Cox17 protein is involved in cisplatin transport to mitochondria and leads to cisplatin’s overall cytotoxicity (35). DNA ligase 1, also known as LIG1, has been singled out as a potentially fruitful therapeutic modification target for ovarian cancer (36). POLE3 supports epigenetic integrity and H3-H4 chaperone activity at the replication fork. WI/SNF gene mutations cause all cancers. GTF2H1 levels affect SWI/SNF-deficient cells’ sensitivity to cisplatin and UV damage (37). As a result, GTF2H1 may be an important prognostic indicator of platinum drug susceptibility in SWI/SNF-deficient cancer cells. Adenylate kinase (AK), which interconverts two adenine nucleotides into stoichiometric quantities of ATP and AMP, performs a crucial function in buffering adenine nucleotides across the tail to sustain flagellar movements. Martin Frejno et al. reported that AK1 inhibits cytarabine and that elevated levels of AK1 correlate with poor survival rates for patients with acute myeloid leukemia treated with Cytarabine (38). Research in this study found that AK1 was closely related to SKCM prognosis. Despite this, the exact mechanism of action is yet to be determined in SKCM. Hence, more research is needed into the role of AK1 in SKCM pathophysiology.

DNA damage repair is linked to immune cell activation in several types of cancer. Chatzinikolaou et al. found a direct connection between DNA damage and innate immune signaling (39). Researchers have discovered that inhibiting PARP and CHK1 increases the number of TILs (40). Moreover, Sato’s group discovered that genotoxic stress, including PARP suppression or irradiation, might upmodulate PD-L1 expression *via* the ATM-ATR/CHK1 pathway (41). According to Jiao et al. (42), PD-L1 may be upregulated by PARP suppressors, resulting in immunological suppression. Based on their study, Garsed et al. identified mutations in the DDR pathways as the cause of immune cell activation and infiltration (43). There has also been evidence that DNA repair mutations and immunological regulation genes contribute to bladder cancer (44). Furthermore, evidence has shown that DDR gene mutations that induce loss of function are common in metastatic SKCM, which could have an impact on immunotherapy efficacy (45). As a result, we conducted this bioinformatics research to investigate the possible link between DDR and immunological escape. According to GSEA functional enrichment analysis, the low-risk cohort was enriched in pathways associated with DDR and immunological modulation, suggesting immunomodulation was associated with a better prognosis. In this research, immune evasion genes were discovered to be overexpressed in SKCM patients who were at a reduced risk. We hypothesized that low-risk patients could derive benefits from immunotherapy because antibodies against immunological escape genes have been shown to enhance the responses of tumor-related T cells to tumor-related antigens, and upregulation of PD-L1 on tumor cells or immune cells has been linked to improved anti-PD1/PD-L1 immunotherapy effectiveness. The study of TIICs, which are recognized for stimulating tumor growth and development, is also a significant method for researching the TME of SKCM. As a result of CIBERSORT analysis, lymphocytes and monocytes were found to be elevated in SKCM samples rather than granulocytes. Subsequently, we evaluated the association between the infiltration of TIICs and risk score and discovered that the extent of immune cell infiltration in the high-risk cohort decreased significantly, as did immune-related functions such as modulation of checkpoints, T cell co-inhibition, and inflammation. These results imply that persons at high risk may develop systemic immunosuppression, which may affect their prognosis.

By suppressing anti-tumor immune cell function, the TME supports tumor growth. The immunosuppressive TME is formed by cancer cells, organ-specific niches, and immune cells with immunoregulatory roles. MDSCs, M2 macrophages, and Foxp3+ Treg cells contribute to the immunosuppressive TME. In our results, the level of M2 macrophage infiltration is positively correlated with the risk score. It has been discovered that M2 macrophages, also known as tumor-associated macrophages (TAMs), serve as immunosuppressive cells in TME. It has been hypothesized that the infiltration of M2 macrophages occurred at an increased level in the low-risk SKCM samples. The M2 macrophages have been shown to express PD-L1, and produce immune-suppressive enzymes, chemokines, and cytokines, thus aiding SKCM tumor angiogenesis and metastasis (46). The level of M1 macrophage, CD8+T cell, infiltrations, on the other hand, is negatively connected with the risk score. It’s worth mentioning that the link between higher riskscores and more infiltrative immune cells needs to be investigated further. The proportions of immune cells in TME changed the aggressive phenotypes induced by deregulation of DRRGs, showing that these genes are involved in the process of activation of the immune system response. DNA repair genes were linked to immunological and metastatic signals, as well as SKCM development and onset. Future SKCM research will require extensive TIICs analysis and large-scale sample research. We anticipated that high-risk patients’ cancers may be more responsive to chemotherapy (such as docetaxel) using the GDSC dataset. To improve survival, high-risk patients may take the corresponding chemotherapy after surgery. In the future, it might be necessary to conduct clinical chemotherapeutic trials.

It is important to highlight that our research has certain limitations as well. To begin with, the data utilized only consisted of a few participants, which might lead to selection bias. Second, in the corresponding publicly accessible GTEx and TCGA datasets, the proportion of healthy samples and SKCM samples was substantially distinct, potentially distorting the findings. Therefore, more tumor specimens should be examined in the future. Finally, since this is bioinformatics research premised on public datasets, experimental and clinical investigations are needed to confirm these results.

## Conclusions

Our research discovered a 12-DRRG signature that might be used to forecast prognosis in SKCM patients. As a result of the present research, we propose that risk scores generated by our model could be used to enhance the current clinical staging system and predict clinical outcomes more accurately. However, more research needs to be conducted to verify our findings.

## Data availability statement

The datasets presented in this study can be found in online repositories. The names of the repository/repositories and accession number(s) can be found in the article/**supplementary material**.

## Author contributions

The work was created by LL and LPL. The data analysis was carried out by LL, SM, and GM. The draft was written by LPL and LL. The manuscript was amended and proofread by YC and LL. The manuscript was reviewed by all authors, who approved the final version before submission.

## Funding

This work was supported by grants from the National Natural Science Foundation of China (Grant No. 81900470) and the Basic and Applied Basic Research Foundation of Guangdong Province (2021A1515110216).

## Acknowledgments

We appreciate the free use provided by TCGA and GEO.

## Conflict of interest

The authors declare that the research was conducted in the absence of any commercial or financial relationships that could be construed as a potential conflict of interest.

## Publisher’s note

All claims expressed in this article are solely those of the authors and do not necessarily represent those of their affiliated organizations, or those of the publisher, the editors and the reviewers. Any product that may be evaluated in this article, or claim that may be made by its manufacturer, is not guaranteed or endorsed by the publisher.

## Abbreviations

SKCM, skin cutaneous melanoma; TCGA, the cancer genome atlas; GTEx, genotype-tissue expression; GEO, gene expression omnibus; DRRGs, DNA repair-related genes; DSS, disease-specific survival; TME, tumor microenvironment; C-index, concordance index; m5C, 5-methylcytosine; m6A, N6-methyladenosine; DDR, DNA damage repair; PARP, poly ADP-ribose polymerase; MEK5, MAP kinase-ERK kinase5; TTK, threonine tyrosine kinase; AIM1, absent in melanoma-1; MGMT, methylguanine-dna methyltransferase; LASSO, Least absolute shrinkage and selection operator; KM, Kaplan-Meier; ROC, receiver operating characteristic; AUC, area under the curve; CYT, cytolytic activity score; GSEA, gene set enrichment analysis; PCA, principal component analysis; NES, normalized enrichment score; ssGSEA, single sample gene-set enrichment analysis; TIIC, tumor-infiltrating immune cells; ICB, immune checkpoint blockade; IPS, immunophenoscore; TMB, tumor mutation burden; TILs, tumor-infiltrating lymphocytes; TIME, tumor immune microenvironment; ICIs, immune checkpoint inhibitors; TYMS, thymidylate synthase; HAX1, HCLS1-associated protein X-1; Cox17, cytochrome c oxidase assembly protein 17; LIG1, ligase 1; AK, adenylate kinase; TAMs, tumor-associated macrophages.
